# Anti-Inflammatory and Skin-Moisturizing Effects of a Flavonoid Glycoside Extracted from the Aquatic Plant *Nymphoides indica* in Human Keratinocytes

**DOI:** 10.3390/molecules23092342

**Published:** 2018-09-13

**Authors:** You Ah Kim, Dong Hee Kim, Chae Bin Park, Tae Soon Park, Byoung Jun Park

**Affiliations:** 1 Skin Science Research Institute, Kolmar Korea Co., Ltd., Chungcheongbukdo 28116, Korea; ahyou2@kolmar.co.kr (Y.A.K.); pkm630@kolmar.co.kr (C.B.P.); 2 Traditional Korean Medicine Technology Division, National Development Institute of Korean Medicine, Gyeongsangbuk-do 38540, Korea; kdh83618@naver.com (D.H.K.); taesoon2p@nikom.or.kr (T.S.P.)

**Keywords:** quercetin 3,7-dimethyl ether 4′-glucoside, *Nymphoides indica*, anti-inflammatory, skin moisturizing

## Abstract

*Nymphoides indica*, an aquatic plant, is used as folk medicine in some countries. Our previous study demonstrated that the methanol extract of *N. indica* inhibited the activity of tyrosinases, tyrosine related protein (TRP)1 and TRP2, and microphthalmia-associated transcription factor, as well as the activity of protein kinase A, by effectively inhibiting cyclic adenosine monophosphate. Although the biological activities of *N. indica* extract have been reported, there are no reports on the skin bioactivity of the main compound(s) on human keratinocytes. This study investigated the anti-inflammatory and moisturizing effects of quercetin 3,7-dimethyl ether 4′-glucoside (QDG) isolated from *N. indica*. In brief, ultraviolet B irradiated keratinocytes were pretreated with different concentrations of QDG, and the effects of QDG on various inflammatory markers were determined. QDG significantly inhibited inflammation-related cytokines and chemokines and enhanced the activation of skin barrier factors. Additionally, QDG also attenuated phosphorylation inhibition of the upstream cytokines and nuclear factor-κB expression. These results suggest that QDG isolated from *N. indica* may serve as a potential source of bioactive substances for chronic inflammatory skin diseases.

## 1. Introduction

Keratinocytes express and release inflammatory mediators in response to skin inflammation, and pro-inflammatory cytokines during the progression phase of the inflammatory process [1]. Therefore, keratinocytes play an important role in the pathogenesis of inflammatory skin diseases such as atopic and contact dermatitis [2]. Epidermal cells can also produce substantial amounts of cytokines, constitutively or following activation, strongly supporting the skin’s function as an immune organ. The cytoplasm of keratinocytes in all the epidermal layers contains the pro-inflammatory cytokine interleukin (IL)-1, and the passively released IL-1 induces the expression of other cytokines such as IL-6, IL-8, IL-10, and IL-13. IL-8 is a chemokine with strong chemotactic effects on polymorphonuclear neutrophils and lymphocytes. Importantly, keratinocytes are known to express IL-8 after stimulation with IL-1β, interferon (IFN)-γ, or tumor necrosis factor (TNF)-α [3].

Additionally, thymus- and activation-regulated chemokine (TARC) and macrophage-derived chemokine (MDC) can play an important role in the development of chronic inflammatory diseases. Keratinocytes and other skin-resident cells produce cytokines that regulate intercellular communication. Thus, limited cytokine expression can contribute to dysfunctional barriers observed in chronic inflammatory diseases [4]. Since chronic inflammatory disease is often characterized by dry, itchy patches, hyaluronan (HA) has been suggested as a useful pharmacological target for its control. Generally, HA’s biological function involves water retention and maintenance of intercellular space. The hypothesized roles for HA in the skin include providing moisture and elasticity, maintaining the dermal structure, and facilitating the transport of ion solutes and nutrients. Thus, HA is suggested as a relevant pharmacological target for the control of chronic inflammatory disease.

Nuclear factor (NF)-κB, an important nuclear transcription factor, initiates the transcription of genes involved in the inflammation and immune responses. Thus, inhibition of NF-κB activity has therapeutic effects in inflammatory diseases [5]. In addition, ultraviolet B (UVB) and pro-inflammatory mediators activate NF-κB by promoting mitogen-activated protein kinase (MAPK) pathways, such as the p38 pathway and the extracellular signal-regulated kinase (ERK) pathway [6,7]. The p38 and ERK pathways are known to mediate cell growth, proliferation, and survival. In addition, the ERK pathway is involved in cellular responses to DNA damage [8].

**Figure 1:**
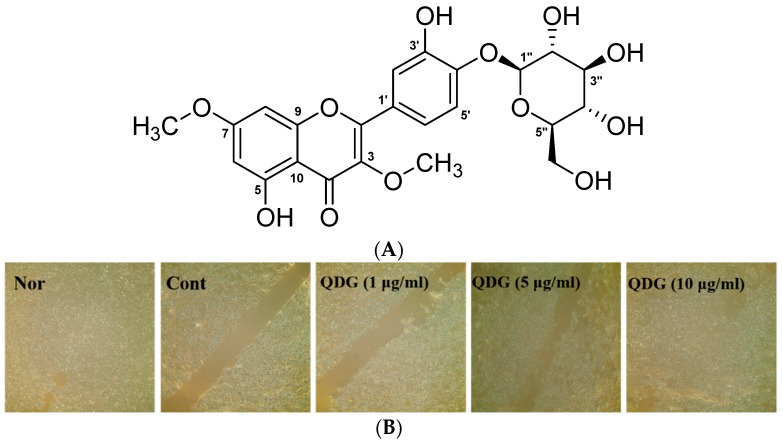
Chemical structure of quercetin 3,7-dimethyl ether 4′-glucoside (QDG) (**A**) and increased cell proliferation and migration activities of QDG-treated human keratinocytes (HaCaT) cells (**B**). HaCaT cells were scratched using a yellow tip. Migration levels of HaCaT cells were observed using an optical microscope and photographs were obtained. HaCaT cells were treated with different concentrations of QDG (1, 5, and 10 μg/mL) for 24 h. QDG treatment leads to an increase in migration of HaCaT cells. Nor: No treatment cell group (0 h), Cont: 20 mJ/cm^2^ ultraviolet B (UVB) treatment cell group, QDG: QDG treatment group.

Natural products isolated from plant sources are responsible for the variety of pharmacological activities. Available reports indicate that many flavonoids have anti-oxidative, hepatoprotective, antibacterial, antiviral, anticancer, anti-inflammatory and anti-photoaging activity [9,10,11,12,13]. In addition, compounds found in plants are known to protect ultraviolet-induced damage to human cells. Genistein, a potent antioxidant, has also been shown to inhibit UVB-induced skin cancer [14,15]. Flavonoid glycosides are also considered to be efficacious compounds of functional ingredients [9,16,17]. Previous studies reported that flavonoid derivative such as quercetin 3-*O*-glucoside, quercetin-7-*O*-β-d-glucopyranoside possesses antioxidant, anti-inflammatory, and wound healing activity [18,19]. Quercetin 3,7-dimethyl ether 4′-glucoside (QDG, Figure 1A) is isolated from the whole plant of *Nymphoides indica* (L.) Kuntze, a perennial rhizomatous free floating-leaved aquatic plant. *N. indica* is traditionally used in the treatment of dysentery, scabies, snake bites, and jaundice. It has also been used for antipyretic, anticonvulsant, aphrodisiac, and antiproliferative purposes. A recent study has reported the pharmacological value of *N. indica* leaves and their phytochemicals due to its antimicrobial, antiprotozoal, anti-oxidant, and antidiabetic activities [20]. Another study demonstrated that the rhizomes of *N. indica* exhibit anticonvulsant activity [21]. Additionally, our previous studies on the biological activities of *N. indica* have demonstrated the inhibitory activity of whole-plant methanol extracts on melanin synthesis [22]. QDG, a major component of the *N. indica* leaves, is reported to have moderate anti-glycation and α-glucosidase inhibitory activities [20]; however, the cosmeceutical effects of QDG, isolated from *N. indica*, on skin cells have not yet been reported.

This study aimed to investigate the anti-inflammatory and skin-moisturizing effects of QDG, isolated from *N. indica,* on immortalized human keratinocytes (HaCaT).

## 2. Results and Discussion

### 2.1. Cell Migration

We confirmed the anti-inflammatory activity in the HaCaT cells of the *N. indica* extract prior to these experiments. As a result, COX-2 protein expression was inhibited by 25%, 38%, and 63% in a concentration-dependent manner at the concentrations of 5, 10, and 20 μg/mL of the *N. indica* extract. In addition, the anti-inflammatory activity of the ethyl acetate fraction (80% at 20 μg/mL) was confirmed by measuring the anti-inflammatory activity of the solvent fraction (data not shown). Therefore, the QDG of this study was isolated from the ethyl acetate fraction and the anti-inflammatory effect of UVB in the HaCaT cells was examined.

The keratinocytes of the skin play an important role in maintaining the homeostasis of the skin by producing various cytokines and growth factors involved in immune and inflammatory reactions and cell proliferation [23]. In this study, the effects of QDG on the migration ability of HaCaT cells were investigated utilizing a wound-healing assay. HaCaT cells, uniformly grown in a monolayer, were scratched with a yellow tip and all the cells in the solid line were removed. The QDG concentration of the keratinocyte layer was determined by the MTT assay and was determined to be 1, 5, and 10 μg/mL (data not shown). Jang et al. [24] reported dibutyryl chitin activity similar to the highest concentration of dibutyryl chitin, 100 μg/mL, and QDG 10 μg/mL, compared with the cell migration of 25, 50, and 100 μg/mL of keratinocytes. QDG was able to confirm the superior cell migration ability. Results indicate that the control group cells showed some migration ability, and the QDG-treated group exhibited a dose-dependent increase in migration. This effect was more pronounced at 10 μg/mL of QDG (Figure 1B). Thus, it can be suggested that QDG provides anti-inflammatory effects by increasing the cell migration ability of keratinocytes.

### 2.2. QDG’s Inhibitory Effect on Cytokine Production

**Figure 2:**
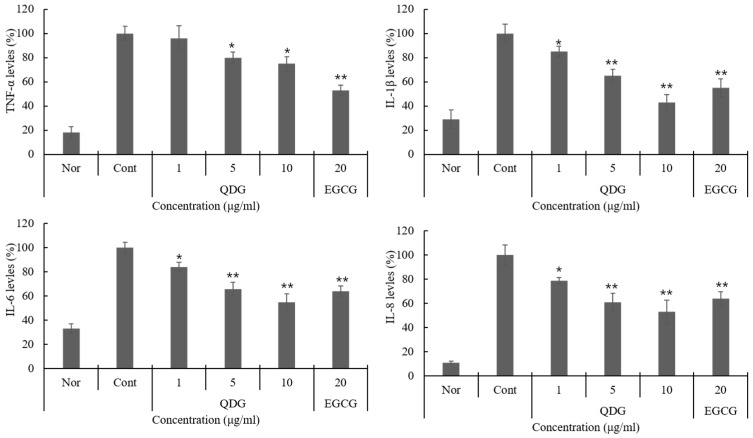
Effect of QDG treatment on cytokine expression in HaCaT cells. HaCaT cells were treated with different concentrations of QDG (1, 5, and 10 μg/mL) after irradiation with 20 mJ/cm^2^ UVB. After 24 h, cytokine expression was determined in the cell supernatant according to the kit manual. Each value represents mean ± SD for the three individual experiments. Nor: No treatment cell group (0 h), Cont: 20 mJ/cm^2^ UVB treatment cell group, QDG = QDG treatment group, EGCG = positive control. *n* = 3, * = *p* < 0.001 and ** = *p* < 0.0001 compared with the control group.

Cytokines function as signaling peptides regulating cell intercourse and providing control of the tissue-specific cell homing. In the skin, chemokines are secreted by the resident cell. Chemokines and cytokines participate in the induction and maintenance of inflammation in the skin [25]. To further understand QDG’s control of the activation of HaCaT cells, we studied its effects on pro-inflammatory cytokines. In the present study, we particularly evaluated the activation of TNF-α, IL-1β, IL-6, and IL-8. Interestingly, QDG dose-dependently suppressed the expression of TNF-α, IL-1β, IL-6, and IL-8. Furthermore, at a dose of 10 μg/mL, QDG significantly inhibited IL-1β, IL-6, and IL-8 (Figure 2). Jeong et al. [26] reported that IL-1β, IL-6, and IL-8 inhibited the cytokine-inhibitory activity of esculetin in HaCaT cells. In particular, QDG showed better IL-1β inhibitory activity. These results demonstrate the potential usefulness of QDG to treat skin inflammation.

### 2.3. QDG’s Inhibitory Effect on Chemokine Production

**Figure 3:**
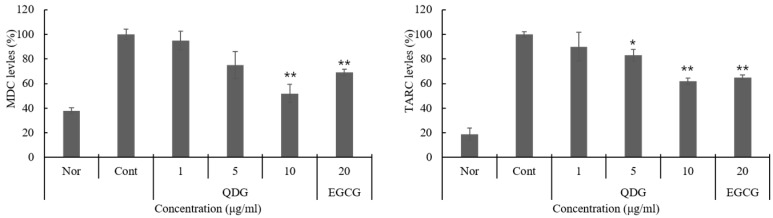
Effect of QDG treatment on macrophage-derived chemokine (MDC) and thymus- and activation-regulated chemokine (TARC) expression in HaCaT cells. HaCaT cells were treated with different concentrations of QDG (1, 5, and 10 μg/mL) after irradiation with 20 mJ/cm^2^ UVB. After 24 h, chemokine expression was determined in the cell supernatant according to the kit manual. Each value represents mean ± SD for the three individual experiments. Nor: No treatment cell group (0 h), Cont: 20 mJ/cm^2^ UVB treatment cell group, QDG = QDG treatment group, EGCG = positive control. *n* = 3. * = *p* < 0.001 and ** = *p* < 0.0001 compared with the control group.

Chronic inflammatory skin diseases such as atopic and contact dermatitis occur due to loss of skin barrier function and inability to control the T helper type 2 (Th2)/T helper type 1 (Th1) immune balance [4,27]. Environmental factors, such as ultraviolet light, are an important factor in inflammatory diseases, with an increase in chemokines and cytokines. Therefore, we explored the effect of QDG on Th2 immune modulation, as well as its effect on the expression of TARC and MDC, members of the CC chemokine subfamily, expressed by the keratinocytes. QDG inhibited UVB-overexpressed MDC and TARC expression in a concentration-dependent manner. Especially at an MDC concentration of 10 μg/mL, the inhibition rate was over 40% higher than that of the control, and it was confirmed that the inhibitory activity was better than that of EGCG (Figure 3). TARC and MDC selectively control the refection and migration of Th2 lymphocytes to inflammatory sites and are considered major factors in the pathogenesis of inflammatory diseases, such as atopic dermatitis [28,29]. Thus, the inhibitory effects of QDG on the expression of these chemokines reveal its potential for the treatment of inflammatory diseases.

### 2.4. QDG’s Effect on the Skin Barrier and Hyaluronic Acid Synthase Production

**Figure 4:**
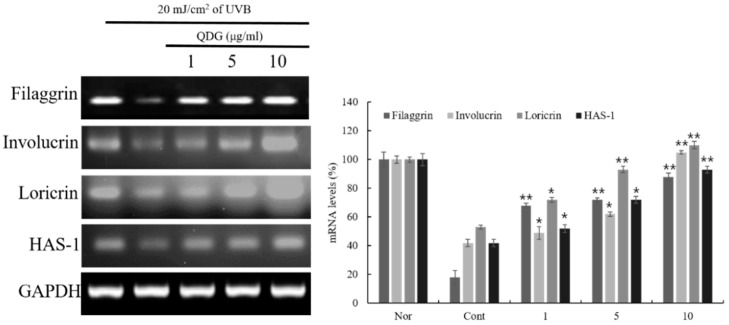
Effect of QDG treatment on skin barrier and hyaluronic acid synthase expressions in HaCaT cells. HaCaT cells were treated with different concentrations of QDG (1, 5, and 10 μg/mL) after irradiation with 20 mJ/cm^2^ UVB. After 6 h, cells were harvested and relative mRNA levels were determined. Histogram shows the densitometry for the skin barrier proteins and hyaluronic acid synthase mRNA normalized to glyceraldehyde 3-phosphate dehydrogenase (GAPDH). Each value represents mean ± SD for the three individual experiments. Nor: No treatment group (0 h), Cont: 20 mJ/cm^2^ UVB treatment group, QDG: QDG treatment group. *n* = 3, * = *p* < 0.001 and ** = *p* < 0.0001 compared with the control group.

The epidermal skin barrier plays a significant role in the susceptibility and severity of chronic inflammatory diseases, such as atopic dermatitis [30,31]. The differentiation of HaCaT cells and the subsequent formation of the skin barrier are a tightly regulated process, often triggered by calcium sensitization and release from the endoplasmic reticulum [32]. This study evaluated the effects of QDG on skin barrier peptide expression and hyaluronic acid production. QDG significantly increased the production of filaggrin, involucrin, loricrin, and hyaluronic acid synthase-1 (HAS-1) with reduced expression rates. In particular, QDG increased the expression of filaggrin, involucrin, loricrin, and HAS-1 by 78%, 85%, 93%, and 95%, respectively, at a final concentration of 10 μg/mL. Interestingly, QDG treatment dose-dependently upregulated the expression of filaggrin, involucrin, loricrin, and HAS-1 levels (Figure 4). Kim et al. [33] reported that compound K enhances the expression of filaggrin and HAS-1 mRNA in the HaCaT cells, and QDG is superior to compound K in the reported skin protection effect. These results suggest that QDG is essential for retaining water and maintaining intercellular space and plays an important role in skin moisture retention by stimulating the expression of genes that facilitate transportation of ions and nutrients.

### 2.5. Phosphorylation of p38/JNK/ERK/IκB

**Figure 5:**
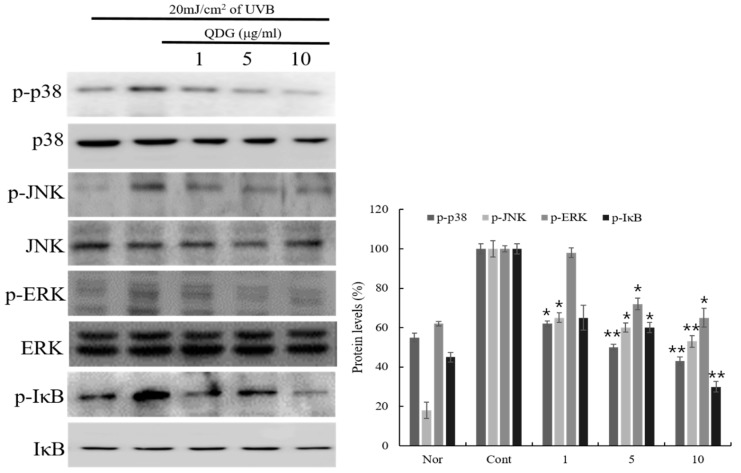
Effects of QDG treatment on mitogen-activated protein kinase (MAPK) phosphorylation expression in HaCaT cells. HaCaT cells were treated with different concentrations of QDG (1, 5, and 10 μg/mL) after irradiation with 20 mJ/cm^2^ UVB. After 30 min, cells were harvested and relative protein levels were determined. Histogram shows the densitometry of phosphorylated-p38, -c-Jun N-terminal kinase (JNK), extracellular signal-regulated kinase (ERK), and inhibitory kappa B alpha (IκBα) proteins normalized to GAPDH. Each value represents mean ± SD for the three individual experiments. Nor: No treatment group (0 h), Cont: 20 mJ/cm^2^ UVB treatment group, QDG: QDG treated group. *n* = 3, * = *p* < 0.001 and ** = *p* < 0.0001 compared with the control group.

Among the inflammatory response intracellular signaling pathways, MAPKs are the well-known signaling pathways involved in the inflammatory response [34,35,36]. Activated MAPKs, in response to cell stimulation, induce the expression of target genes by activating other kinases or transcription factors, such as p38, c-Jun N-terminal kinase (JNK), and ERK. p38 is a central regulator of the inflammatory response regulating IL-6, IL-8, and TNF-α production and the expression of nitric oxide and metalloproteinase [37]. JNK regulates the inflammatory response through c-Jun phosphorylation and increased activator protein (AP)-1 [38]. ERK is extensively activated by stimulating factors, and activated ERK induces the translocation of NF-κB into the nucleus, known to be the main mechanism of inflammatory expression, and regulates inflammatory expression [39]. These transcription factors are closely associated with chemokine and cytokine production in UVB-induced HaCaT cells. The results showed that QDG significantly inhibited p38, JNK, and ERK phosphorylation by 57%, 47%, and 35%, respectively, at a concentration of 10 μg/mL. In addition, QDG further upregulated the expression level of inhibitory kappa B alpha (IκBα) and inhibited the phosphorylation of IκBα (Figure 5). Within the cell, increased expression of p-IκBα results in the degradation of IκBα, an endogenous inhibitor that prevents NF-κB translocation. These results confirmed that QDG exerts its anti-inflammatory effect by controlling expression of inflammatory factor through p38, JNK, and ERK.

### 2.6. Signaling Pathways Leading to the Activation of NF-κB

**Figure 6:**
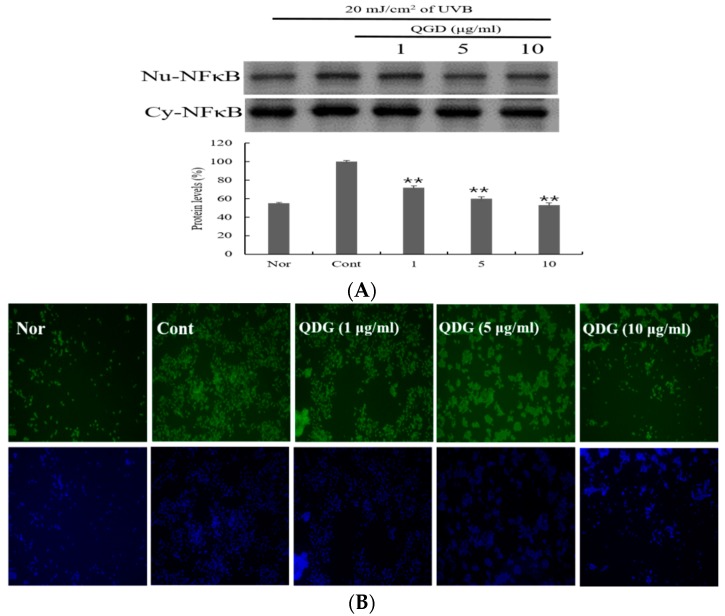
Effect of QDG treatment on NF-κB protein expression in HaCaT cells. HaCaT cells were treated with different concentrations of QDG (1, 5, and 10 μg/mL) after irradiation with 20 mJ/cm^2^ UVB. After 6 h, cells were harvested, and (**A**) protein and (**B**) NF-κB–FITC levels were determined. Histogram shows the densitometry of NF-κB protein normalized to glyceraldehyde 3-phosphate dehydrogenase. Each value represents mean ± SD for the three individual experiments. Nor: No treatment group (0 h), Cont: 20 mJ/cm^2^ UVB treatment group, QDG = QDG treatment group. *n* = 3, * = *p* < 0.001 and ** = *p* < 0.0001 compared with the control group.

The NF-κB signaling pathways have been implicated in the development and progression of chronic inflammation disease. Chronic inflammation disease, such as atopic dermatitis is associated with the activation and expression of pro-inflammatory cytokines [40,41]. The translocation of NF-κB from the cytoplasm to the nucleus is a molecular event associated with inflammation. This process results in the transcription of pro-inflammatory genes that contribute to the progression of the inflammation disease [42,43]. Therefore, we investigated the effect of QDG on UVB-induced NF-κB translocation. QDG showed 83%, 65%, and 57% NF-κB protein expression in 1, 5, and 10 μg/mL concentration, respectively (Figure 6A). QDG showed stronger inhibitory activity when compared to only UVB-irradiated group, a potent pharmacological inhibitor of NF-κB translocation into the nucleus (Figure 6A,B). Interestingly, compounds derived from natural products such as curcumin, capsaicin, resveratrol, and green tea polyphenols have been shown to be potent inhibitors of the NF-κB pathway by inhibiting IKK activity [44,45]. Since QDG could be shown to inhibit NF-κB activation, it can be assumed that QDG affects IKK and thus affects the translocation of NF-κB from cytoplasm into the nucleus. Therefore, QDG is considered similar to the way the previously reported *Rhizoma coptidis* extract affects the NF-κB pathway in HaCaT [46]. This approach has been suggested as an indirect method to control inflammatory disease. These results show that QDG activates molecular events that prevent the translocation of NF-κB.

## 3. Materials and Methods

### 3.1. General Procedures

Column chromatography was conducted using 70–230 mesh silica gel (Merck, Darmstadt, Germany). Watchers^®^ Silica gel Si 60 (70–230 mesh) was used for column chromatography (Isu Industry Co., Seocho, Korea). TLC analysis was carried out on precoated silica gel 60 F254 plates (Merck). Detection of spots on the TLC plate was performed by observation under a UV lamp (Spectroline, model CM-24A, Spectronics Corp., New York, NY, USA) or by spraying 10% aqueous H_2_SO_4_ on the developed plate followed by heating. Prep LC was performed with a YMC LC-Forte/R (YMC, Kyoto, Japan). NMR spectra were recorded on a Bruker Ascend 400 and Avance 500 (Bruker, Rheinstetten, Germany). High-resolution electrospray ionization mass spectrometry (HR-ESI-MS) was carried out using a SYNAPT G2 electrospray mass spectrometer (Waters, Elstree, UK) at the Korean Basic Science Institute, Seoul, Korea.

### 3.2. Reagents

Dulbecco’s Modified Eagle Medium (DMEM), fetal bovine serum (FBS), and streptomycin‒penicillin were purchased from GIBCO (Grand Island, NY, USA). The recombinant human IL-6, 8 recombinant human TNF-α, recombinant human IFN-γ, Quantikine ELISA kits for Macrophage Derived Chemokine (MDC), Thymus Activation Regulated Chemokine (TARC) were purchased from R&D Systems (Minneapolis, MN, USA). The primary antibodies for involucrin, loricrin, filaggrin p38, JNK, and ERK were purchased form Abcam (Cambridge, UK). Antibodies against nuclear factor κB (NF-κB), inhibitory kappa B alpha (IκBα), and phosphorylated IκBα were purchased from Cell Signaling (Beverly, MA, USA). Mouse monoclonal anti-β-actin was obtained from Sigma Aldrich (St. Louis, MO, USA). Polyvinylidene difluoride (PVDF) membrane, Tetramethylethylenediamine (TEMED), Sodium Dodecyl Sulfate (SDS) and acrylamide were purchased from Bio-rad (Hercules, CA, USA). Nuclear and cytoplasmic extraction reagents and first strand cDNA synthesis kit were obtained from Thermo Scientific (Rockford, IL, USA). All other chemical reagents were of the highest pure analytical grade commercially available.

### 3.3. Plant Material

Whole plants of *Nymphoides indica* (L.) Kuntze were purchased from Agricultural Corporation Lotus Green Co., Ltd. in Gwangju, Korea in May, 2016. The plant taxa were identified using the DNA barcoding system, by comparing the sequences obtained either with public databases (NBCI GenBank) and/or with a database made for this purpose from the National Institute of Biological Resources (NIBR), Korea (voucher No. NIBRVP0000592689).

### 3.4. Isolation and Structure Determination of *Compound **1***

**Figure 7:**
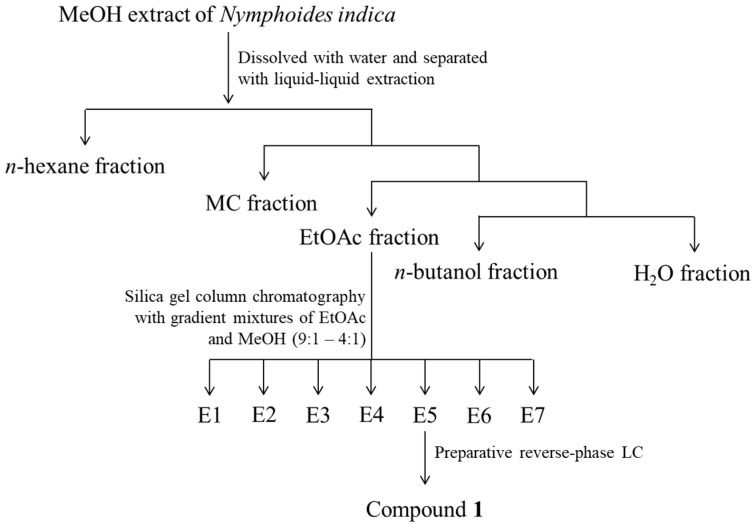
Separation procedure of methanol extract from *Nymphoides indica*.

The air-dried samples (700 g) were cut into pieces and extracted for three days with 95% methyl alcohol (MeOH) (18 L × 3) at room temperature. The combined crude extracts were concentrated under reduced pressure to yield a MeOH extract (159.8 g) and dissolved in distilled water (H_2_O). The suspended extract was partitioned using *n*-hexane, methylene chloride (MC), ethyl acetate (EtOAc), and *n*-butanol to yield layers of 14.5, 2.4, 3.7, and 12.0 g, respectively. A portion of EtOAc fraction (3.0 g) was subjected to silica gel column chromatography with gradient mixtures of EtOAc and MeOH (9:1–4:1) to give seven fractions (Fr. E1–Fr. E7). Fr. E5 was subjected to preparative reverse-phase LC (YMC Actus Triart C18 column; 250 × 20 mm, S-5 μm, 12 mm; flow rate, 10.0 mL/min; 30% acetonitrile in H_2_O for 60 min; UV detection at 254 nm) to afford compounds **1** (167 mg) (t_R_ = 45.0 min) (Figure 1A and Figure 7).

*Quercetin 3,7-dimethyl ether 4′-glucoside*: Yellow power, C_23_H_24_O_12_ (mol. wt. 492); HR-ESI-MS (positive ion mode) *m*/*z* 493.1346 [M + H]^+^, ^1^H-NMR (CD_3_OD, 400 MHz): δ 7.63 (1H, *d*, J = 2.0 Hz, H-2′), 7.59 (1H, *dd*, J = 2.0, 8.4 Hz, H-6′), 7.31 (1H, *d*, J = 8.4 Hz, H-5′), 6.57 (1H, *d*, J = 2.0 Hz, H-8), 6.31 (1H, *d*, J = 2.0 Hz, H-6), 4.94 (1H, *d*, J = 7.2 Hz, H-1″), 3.4–3.8 (6H, *m*, protons of sugar party), 3.88 (3H, *s*, 3-OCH_3_), 3.81 (3H, *s*, 7-OCH_3_); ^13^C-NMR (CD_3_OD, 100 MHz): 180.3 (C-4), 167.5 (C-7), 163.0 (C-5), 158.5 (C-9), 157.6 (C-2), 149.4 (C-4′), 148.4 (C-3′), 140.5 (C-3), 126.6 (C-1′), 122.1 (C-6′), 117.9 (C-5′), 117.4 (C-2′), 107.0 (C-10), 103.5 (C-1″), 99.2 (C-6), 93.3 (C-8), 78.6 (C-5″), 77.7 (C-3″), 75.0 (C-2″), 71.5 (C-4″), 62.3 (C-6″), 60.8 (3-OCH_3_), 56.7 (7-OCH_3_): Supporting information [20,47].

### 3.5. Cell Culture and UVB Irradiation

Immortalized human keratinocytes (HaCaT) were purchased from the American Type Culture collection (Manassas, VA, USA). The cells were cultured in high-glucose DMEM containing 10% FBS, 1% streptomycin‒penicillin at 37 °C in a 5% CO_2_ humidified atmosphere. The cells were exposed to UVB radiation using an UV irradiation system (BIO-LINK Crosslinker, WA, Wembley, Australia) delivering the 280–320 nm wavelength range, with maximum emission at 312 nm. Seeded cells were rinsed with PBS and then exposed to 20 mJ/cm^2^ of UVB.

### 3.6. Cell Migration

HaCaT cells were incubated, at 5 × 10^5^ cells/mL for 24 h, in a cell culture incubator. Next, the cell monolayers were scratched with a 200-μL yellow tip and washed once with phosphate-buffered saline (PBS). Next, cell monolayers were treated with different concentrations of QDG (1, 5, and 10 μg/mL) and cultured in a CO_2_ incubator for 24 h. Cell motility was assessed 24 h later, using a photomicroscope, and the scratched area was measured. Measurements were taken to determine the distance traveled, in the 24 h period, by measuring the scratched area in the photographed pictures.

### 3.7. Immunoassays for Cytokines and Chemokines

HaCaT cells (1 × 10^5^ cells/300 μL or 5 × 10^5^ cells/400 μL for the cytokine or chemokine assay, respectively) were grown in a 24-well plate and treated with UVB (20 mJ/cm^2^). After centrifugation at 412× *g* for 10 min, the amounts of TNF-α, IL-1β, IL-6, IL-8, MDC and TARC in the culture supernatant were analyzed using the corresponding enzyme-linked immunosorbent assay (ELISA) kits, according to the manufacturer’s instructions. The absorbance was measured at 450 nm using a microplate reader (Magellan; Tecan Ltd, Salzburg, Austria).

### 3.8. Measurement of Skin Barrier Peptide and Hyaluronic Acid

HaCaT cells were seeded in six-well plates, at a density of 1 × 10^5^ cells/well. The cells were starved for 24 h, after which they were stimulated with 1, 5, and 10 μg/mL of QDG for 24 h. Supernatants were collected and ELISA kits utilized to measure relative filaggrin, loricrin, and HA production, according to the manufacturer’s instruction.

### 3.9. Preparation of Cytosolic and Nuclear Extracts

HaCaT cells (5 × 10^6^ cells/mL) were treated with LPS for 30 min, at 37 °C. Keratinocyte cytosolic and nuclear extracts were prepared as previously described [48]. Keratinocytes were harvested by centrifugation at 412× *g* for 10 min and washed twice with PBS. The cells were suspended in 400 μL of lysis buffer (10 μM KCl, 1.5 μM MgCl_2_, 0.1 μM EDTA, 0.1 μM EGTA, 1 μM dithiothreitol, 0.5 μM PMSF, 1 μM sodium orthovanadate, 2 μg/mL aprotinin, 2 μg/mL leupeptin, and 10 mM Hepes-KOH, pH 7.8) and were allowed to swell on ice for 15 min. Next, 25 μL of a 10% Nonidet NP-40 solution (final concentration: approximately 0.6%) were added, and the tubes were vigorously vortexed for 10 s. The homogenates were centrifuged at 12,000× *g* for 10 min at 4 °C. The supernatants were stored as cytoplasmic extracts and kept at −70 °C. The nuclear pellets were re-suspended in 50 μL of an ice-cold hypertonic solution containing 5% glycerol and 0.4 M NaCl lysis buffer. Furthermore, the tubes were incubated on ice for 30 min and then centrifuged at 12,000× *g* for 15 min at 4 °C. The supernatants were collected as nuclear extracts and stored at −70 °C. Protein concentrations were determined using the Bradford method according to the manufacturer’s instructions (Bio-Rad Laboratories).

### 3.10. Western Blot Assay

HaCaT cells were collected on ice, washed three times with ice-cold PBS, and treated with a homogenizing buffer containing protease inhibitor cocktail (Roche Diagnostics, Indianapolis, IN, USA). After brief sonication, the cell lysates were centrifuged at 12,000 rpm for 10 min, and supernatants were collected. Next, the protein concentrations were determined using Bradford protein assay reagent (Bio-Rad Laboratories). Twenty micrograms of the protein were separated on a 7.5–10% SDS gel and then transferred to a PVDF membrane, which was then probed with specific primary antibodies overnight with gentle shaking, followed by incubation with secondary antibodies for 1 h. Blots were developed using enhanced chemiluminescence (Amersham Biosciences, Little Chalfont, Buckinghamshire, UK) and quantified using a Gel-pro analyzer (Media Cybernetics Inc., Rockville, MD, USA).

### 3.11. Immunofluorescence

HaCaT cells were aliquoted in an eight-well Lab-Tek chamber (Nalge-Nunc, Madison, WI, USA) with 1 × 10^3^ cells and allowed to grow for 24 h after QDG treatment. Next, they were washed with cold PBS three times and 95% Triton X-100 was added for 10 min. After washing with PBS, 1% of bovine serum albumin was added, and the cells were incubated for 1 h. Next, the c-fos primary antibody (1:100) was added, and the cells were incubated at 4 °C overnight. In the next step, cells were treated with a secondary antibody, Alexa 488-conjugated goat anti-mouse immunoglobulin G (Invitrogen, Thermo Fisher Scientific, Waltham, MA, USA), and fluorescein isothiocyanate (1:1000). Stained cells were then mounted on a slide after washing with PBS and observed by a fluorescent microscope for NF-κB activity.

### 3.12. Statistical Analysis

Analysis of variance was performed in SPSS (SPSS Inc., Chicago, IL, USA). All data are expressed as mean ± SD, and statistically significant differences between experimental and control values were analyzed by one-way ANOVA followed by a *t*-test. * *p*-value < 0.001, ** *p*-value < 0.0001 was considered statistically significant.

## 4. Conclusions

We investigated the effects of QDG, a *Nymphoides indica* component, against the activity of inflammation-related factors in UVB-irradiated keratinocytes. QDG inhibited TNF-α, IL-1β, IL-6, and IL-8 in a dose-dependent manner and also inhibited the expression of TARC and MDC induced by UVB irradiation. In the UVB-exposed group, the expression of filaggrin, involucrin, loricrin, and HAS-1 was decreased compared with normal cells. We also investigated whether the anti-inflammatory effects of QDG are due to its modulation of NF-κB. QDG treatment in keratinocytes not only reduced the phosphorylation of p38, JNK, and ERK in a concentration-dependent manner, but also decreased NF-κB levels and the generation of chronic inflammatory disease factors due to UVB irradiation. According to the report, glycolic acid induced by UVB stimulation inhibits overexpressed factors by UVB-mediated NF-κB signaling in the HaCaT cells, suggesting that it inhibits inflammation [49]. Our study also suggests that QDG has an anti-inflammatory effect on the overexpression of inflammatory factors by UVB stimulation. Taken together, these findings suggest that QDG may be useful for the treatment of chronic inflammatory skin diseases.
